# Mislocalization of XPF-ERCC1 Nuclease Contributes to Reduced DNA Repair in XP-F Patients

**DOI:** 10.1371/journal.pgen.1000871

**Published:** 2010-03-05

**Authors:** Anwaar Ahmad, Jacqueline H. Enzlin, Nikhil R. Bhagwat, Nils Wijgers, Anja Raams, Esther Appledoorn, Arjan F. Theil, Jan H. J. Hoeijmakers, Wim Vermeulen, Nicolaas G. J. Jaspers, Orlando D. Schärer, Laura J. Niedernhofer

**Affiliations:** 1 Department of Microbiology and Molecular Genetics, University of Pittsburgh School of Medicine, Pittsburgh, Pennsylvania, United States of America; 2 University of Pittsburgh Cancer Institute, Pittsburgh, Pennsylvania, United States of America; 3 Institute of Molecular Cancer Research, University of Zurich, Zurich, Switzerland; 4 Department of Human Genetics, University of Pittsburgh School of Public Health, Pittsburgh, Pennsylvania, United States of America; 5 Department of Cell Biology and Genetics, Erasmus Medical Center, Rotterdam, The Netherlands; 6 Departments of Pharmacological Sciences and Chemistry, Stony Brook University, Stony Brook, New York, United States of America; University of Washington, United States of America

## Abstract

Xeroderma pigmentosum (XP) is caused by defects in the nucleotide excision repair (NER) pathway. NER removes helix-distorting DNA lesions, such as UV–induced photodimers, from the genome. Patients suffering from XP exhibit exquisite sun sensitivity, high incidence of skin cancer, and in some cases neurodegeneration. The severity of XP varies tremendously depending upon which NER gene is mutated and how severely the mutation affects DNA repair capacity. XPF-ERCC1 is a structure-specific endonuclease essential for incising the damaged strand of DNA in NER. Missense mutations in *XPF* can result not only in XP, but also XPF-ERCC1 (XFE) progeroid syndrome, a disease of accelerated aging. In an attempt to determine how mutations in *XPF* can lead to such diverse symptoms, the effects of a progeria-causing mutation (XPF^R153P^) were compared to an XP–causing mutation (XPF^R799W^) *in vitro* and *in vivo*. Recombinant XPF harboring either mutation was purified in a complex with ERCC1 and tested for its ability to incise a stem-loop structure *in vitro*. Both mutant complexes nicked the substrate indicating that neither mutation obviates catalytic activity of the nuclease. Surprisingly, differential immunostaining and fractionation of cells from an XFE progeroid patient revealed that XPF-ERCC1 is abundant in the cytoplasm. This was confirmed by fluorescent detection of XPF^R153P^-YFP expressed in *Xpf* mutant cells. In addition, microinjection of XPF^R153P^-ERCC1 into the nucleus of XPF–deficient human cells restored nucleotide excision repair of UV–induced DNA damage. Intriguingly, in all *XPF* mutant cell lines examined, XPF-ERCC1 was detected in the cytoplasm of a fraction of cells. This demonstrates that at least part of the DNA repair defect and symptoms associated with mutations in *XPF* are due to mislocalization of XPF-ERCC1 into the cytoplasm of cells, likely due to protein misfolding. Analysis of these patient cells therefore reveals a novel mechanism to potentially regulate a cell's capacity for DNA repair: by manipulating nuclear localization of XPF-ERCC1.

## Author Summary

XPF-ERCC1 is a nuclease that plays a critical role in DNA repair. Mutations in *XPF* are linked to xeroderma pigmentosum, characterized by sun sensitivity, high incidence of skin cancer, and neurodegeneration, or XFE progeroid syndrome, a disease of accelerated aging. Herein we report the unexpected finding that mutations in *XPF* cause mislocalization of XPF-ERCC1 to the cytoplasm. Recombinant mutant XPF-ERCC1 derived from XP– and XFE–causing alleles are catalytically active and if delivered to the nucleus of cells restore DNA repair. This demonstrates that protein mislocalization contributes to defective DNA repair and disease arising as a consequence of mutations in *XPF*. It also illustrates a novel mechanism of regulating a cell's capacity for DNA repair: by manipulating nuclear localization of XPF-ERCC1 to enhance or inhibit repair and to prevent cancer or tumor resistance to chemotherapy, respectively.

## Introduction

Xeroderma pigmentosum (XP) is a rare autosomal recessive disease characterized by photosensitivity and a greater than a 1000-fold increased risk of skin cancer in sun-exposed areas of the skin [1]. In approximately 20% of patients, there is also progressive neurodegeneration leading to loss of coordinated motion, vision and hearing [2],[3]. XP is caused by mutations in genes that encode proteins required for nucleotide excision repair (NER) of DNA. Eight complementation groups of XP have been identified based on fusion studies with XP patient cells. These complementation groups include XP-A through XP-G and a variant, XP-V. The severity of XP varies tremendously, with diagnosis occurring anywhere from infancy to adulthood [1]. The severity of the disease is determined largely by which gene is mutated and to what extent the mutation affects NER.

NER removes helix-distorting lesions in DNA, for example cyclobutane pyrimidine dimers (CPDs) and pyrimidine pyrimidone photoproducts (6–4PPs) caused by the ultraviolet (UV) component of sunlight [4]. There are two ways by which DNA damage is recognized in NER. Lesions anywhere in the genome can be recognized by the complex XPC-RAD23B [5],[6]. For some lesions, this is facilitated by a second complex XPE/DDB2-DDB1 [7]. Alternatively, lesions that occur in the coding strand of DNA, within transcribed regions, can trigger NER if they stall progression of RNA polymerase II [8],[9]. This requires CSA, CSB and XAB2 [10]–[12]. Once the damage is recognized, the subsequent steps of damage excision are believed to be uniform. The basal transcription factor TFIIH is recruited to the site of helix-distortion to unwind the DNA around the lesion, using two of its ten subunits, XPB and XPD [8],[13]. RPA and XPA bind the unwound repair intermediate to stabilize it and recruit subsequent factors. The damaged strand of DNA is then incised by two structure-specific endonucleases, the heterodimer of XPF-ERCC1 and XPG, which cut 5′ and 3′ of the lesion, respectively [14]–[17]. This leads to removal of the lesion as part of a 24–32 base oligonucleotide. The resultant gap is filled by the replication machinery including RPA, PCNA, RF-C, DNA polymerase δ/ε, and the backbone is sealed by DNA LIGI or LIGIIIα-XRCC3 [18]–[20].

XPF-ERCC1 is a highly conserved endonuclease that nicks double-stranded DNA 5′ to a junction with single-stranded DNA [21]. In addition to NER, XPF-ERCC1 is involved in the repair of DNA interstrand crosslinks (ICL) [22] and double-strand breaks [23]. XPF and ERCC1 are paralogs thought to have arisen by gene duplication of the conserved nuclease and helix-hairpin helix protein-interaction domains [24]. The proteins interact via their C-terminal helix-hairpin-helix domains and this interaction is required to stabilize both proteins in vivo [25],[26]. XPF contains the nuclease catalytic domain [27], whereas ERCC1 mediates the interaction with XPA and recruitment to NER complexes [28],[29]. Unlike NER-specific proteins XPA and XPC, XPF-ERCC1 appears to be essential for human development or viability since no patients have yet been identified who are homozygous for early nonsense or frameshift mutations in either gene.

XP-A and XP-C are among the most common complementation groups in XP [30]. XP-C patients have severe skin abnormalities but generally lack neurological symptoms [31]. In contrast, XP-A patients show profound neurodegeneration, in addition to cutaneous features [1]. XP-F patients typically have very mild cutaneous features of XP, including late onset of skin cancer, but often have complications due to neurodegeneration as adults. It was recently discovered that mutations in *XPF* can lead to a second disease, XFE progeroid syndrome (short for XPF-ERCC1), characterized by spontaneous, accelerated aging of multiple tissues, including the nervous system [32]. Herein, we attempted to understand the molecular basis for how mutations in *XPF* could lead to such diverse outcomes. This led to the surprising discovery that mutation of *XPF* promotes mislocalization of XPF-ERCC1 to the cytoplasm of cells.

## Results

### Characterization of R^153^P-XPF-ERCC1 activity in vitro

**Figure 1:**
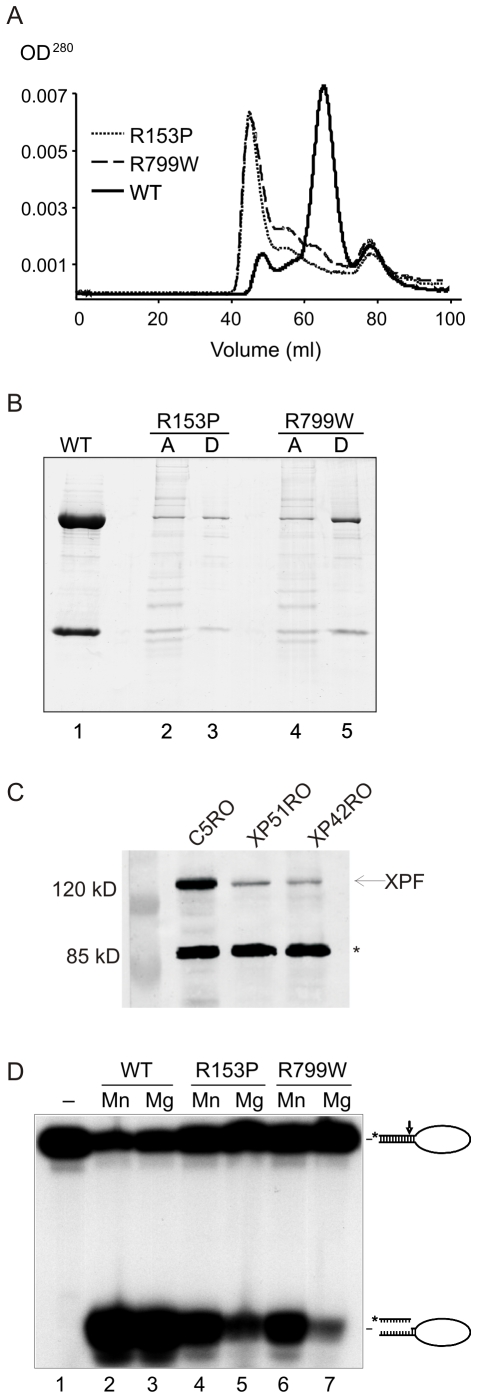
Biochemical characterization of XPF^R153P^-ERCC1 and XPF^R799W^-ERCC1 mutants. (A) Gel filtration profiles from the purification of recombinant XPF-ERCC1, XPF^ R153P^-ERCC1 and XPF^ R799W^-ERCC1 from baculovirus-infected Sf9 insect cells using a His_6_ tag on ERCC1. Aggregated proteins elute at ∼45 ml in the void volume of the column; heterodimeric XPF-ERCC1 elutes at ∼65 ml, corresponding to ∼200 kD, and monomeric ERCC1 elutes at ∼78 ml (∼50kD). (B) SDS PAGE analysis of purified protein complexes. Lane 1, 3 and 5 (D): XPF-ERCC1, XPF^ R153P^-ERCC1 and XPF^ R799W^-ERCC1, respectively, after purification over NTA-agarose, gel filtration and heparin columns. Lanes 2 and 4 (A) show the proteins present in the fractions eluting at 45 ml in the gel filtration column step of XPF^ R153P^-ERCC1 and XPF^ R799W^-ERCC1, respectively. (C) Immunodetection of XPF in normal (C5RO) and *XPF* mutant cells. The star indicates the migration of a cross-reactive band demonstrating equal loading [32]. (D) Incision activities of XPF-ERCC1, XPF^ R153P^-ERCC1 and XPF^ R799W^-ERCC1 (200 fmol) on a 5′-^32^P-labeled stem-loop DNA substrate (100 fmol) in the presence of either 0.4 mM MnCl_2_ (lanes 2, 4 and 6) or 2 mM MgCl_2_ (lanes 3, 5 and 7). Reactions were analyzed on a 15% denaturing polyacrylamide gel. The 46-mer substrate and 9–10-mer products are indicated.

We first asked if mutations in *XPF* that cause mild or severe disease differentially affect the biochemical properties of XPF-ERCC1. To answer this, we compared the biochemical properties of XPF-ERCC1 from two patients, XP42RO (a patient with mild XP, homozygous for a mutation causing an R^799^W substitution in *XPF* [33]) and XP51RO (a patient with XFE progeroid syndrome, homozygous for a mutation causing an R^153^P substitution in *XPF* [32]) to that of wild type XPF-ERCC1. Recombinant XPF^WT^-ERCC1, XPF^R153P^-ERCC1 and XPF^R799W^-ERCC1 were purified from baculovirus-infected Sf9 insect cells using a His_6_ tag on ERCC1. We previously reported [27] that our purified preparations of XPF^WT^-ERCC1 elute from a gel-filtration column in three fractions: (1) a minor fraction in the void volume (∼45 ml) containing aggregated, inactive protein; (2) active heterodimeric XPF-ERCC1 at ∼65 ml, which corresponds to a molecular weight of ∼200 kD, as expected, and (3) monomeric ERCC1, which peaks at ∼78 mL, which corresponds to ∼50 kD. Recombinant XPF^WT^-ERCC1 eluted as expected (Figure 1A). Both mutant protein complexes eluted with similar profiles that differed substantially from that of XPF^WT^-ERCC1. The majority of the mutant complexes eluted at ∼45 mL rather than at 65 ml, indicating that they were aggregated. The peak at 78 ml, corresponding to free ERCC1 was identical for both mutant and WT XPF-ERCC1 preps. These results suggest that the mutations in *XPF* that cause both mild and severe disease lead to protein misfolding that does not interfere with ERCC1 binding, but does lead to protein aggregation.

We were able to purify a small amount of XPF^R153P^-ERCC1 and XPF^R799W^-ERCC1 from the fractions eluting at 65 ml, indicating that at least some of the mutant proteins are likely to be properly folded. SDS-PAGE analysis of the complexes after an additional purification step over a heparin column revealed dramatically reduced yields of the complexes of XPF^R153P^-ERCC1 and XPF^R799W^-ERCC1 compared to XPF^WT^-ERCC1 (Figure 1B). Similarly, the amount of XPF protein detectable by immunoblot in whole cell extracts of human fibroblasts harboring the XPF^R153P^ and XPF^R799W^ mutations (XP51RO and XP42RO, respectively) was reduced compared to normal cells (C5RO) (Figure 1C).

The catalytic activity of the purified heterodimers was investigated by measuring their ability to incise a ^32^P-end-labeled stem–loop DNA substrate at the single-strand:double-strand DNA junction in the presence of 0.4 mM MnCl_2_ or 2 mM MgCl_2_ at a 2-fold molar excess of protein over substrate (Figure 1D). With XPF^WT^-ERCC1, >80% of the stem-loop substrate was cleaved. Both XPF^R153P^-ERCC1 and XPF^R799W^-ERCC1 also incised the DNA substrate, demonstrating that both mutant complexes retain catalytic activity (Figure 1D, lanes 5 & 7). Incision by both mutant complexes was reduced compared to the WT complex. This may simply reflect the fact that preparations of mutant heteroduplexes were less concentrated than XPF^WT^-ERCC1 (Figure 1B), inevitably leading to differences in the buffering conditions between incision reactions.

We previously observed that mutant XPF-ERCC1 complexes tend to be more active in the presence of Mn^2+^ than Mg^2+^ since this metal has less stringent requirements for the proper alignment of the active site residues [27]. Consistent with this, incision by XPF^R153P^-ERCC1 and XPF^R799W^-ERCC1 was increased ∼2-fold in the presence of Mn^2+^ compared to Mg^2+^, whereas the cation had no effect on incision by XPF^WT^-ERCC1 (Figure 1D). These data support the conclusion that even monomeric XPF^R153P^ and XPF^R799W^ are to some extent misfolded. Notably, there was not a dramatic difference in the enzymatic activity of XPF^R153P^-ERCC1 and XPF^R799W^-ERCC1 on stem-loop DNA substrates, indicating that the biochemical basis for the more severe phenotype associated with the R^153^P mutation is unlikely to be simply a loss of catalytic activity.

### Immunolocalization of XPF protein

**Figure 2:**
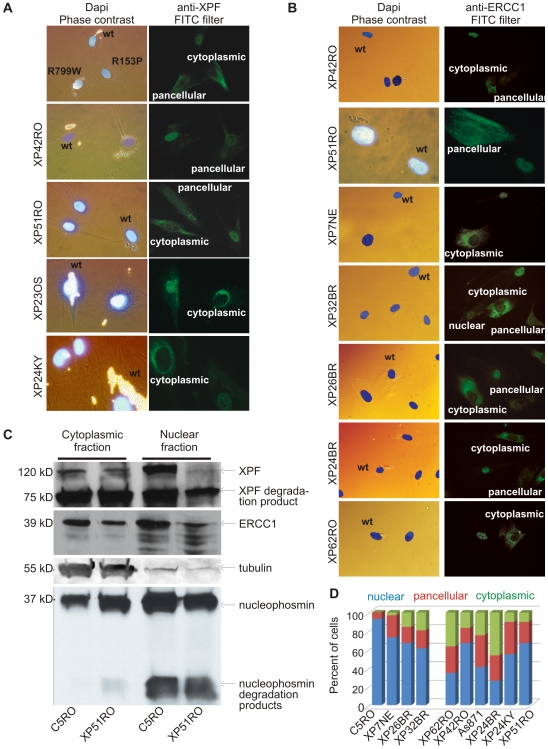
Differential immunofluorescence of cells from patients with *XPF* mutations. Fibroblasts from patients with mutations in *XPF* and a normal control were grown in the presence of different size beads. After 24 hr the cultures were washed to remove extracellular beads, mixed and co-plated on glass coverslips. The next day, the cells were fixed and immunostained as indicated. Cells were stained with Dapi to identify nuclei and examined by phase contrast microscopy to identify the cell type by their bead content and by fluorescence microscopy for immunodetection of XPF or ERCC1. (A) Analysis of XPF protein sub-cellular localization. Cells from an unaffected individual were labeled with 2 µM beads; *XPF* mutant cells were labeled with 0.8 µM beads. (B) Analysis of ERCC1 subcellular localization in patients with mutations in *XPF*. (C) Immunodetection of XPF and ERCC1 in nuclear and cytoplasmic fractions of normal fibroblasts (C5RO) and *XPF* mutant cells (XP51RO). Tubulin is used as a loading control of the cytoplasmic fraction. Nucleophosmin is used as a loading control for the nuclear fraction. (D) Quantitation of the fraction of cells containing exclusively nuclear XPF-ERCC1, XPF-ERCC1 in the nucleus and cytoplasm (pancellular) or exclusively cytoplasmic complex, as determined from immunofluorescence images (n≥100 cells per cell line).

R^153^P is situated in a lysine-rich domain of XPF that might be part of a complex nuclear localization sequence (NLS). Thus we next asked if XPF^R153P^-ERCC1 is mislocalized in cells. Differential immunofluorescence was used to identify the subcellular localization of XPF-ERCC1 in *XPF* mutant cell lines that were co-cultured with normal cells as an internal control [34]. XPF was detected exclusively in the nucleus of normal fibroblasts (Figure 2A, upper panel). In the same sample, XPF was detected in the cytoplasm of XP51RO cells (harboring XPF^R153P^). In XP42RO cells (harboring XPF^R799W^), the XPF signal was pancellular so that the nucleus and cytoplasm could not be distinguished from one another. To explore this further, we used immunofluorescence to detect XPF in additional XP-F patient cell lines and to our surprise discovered that in all mutant cell lines XPF was frequently detected in the cytoplasm. In all cases, *XPF* mutant cell lines could be discriminated from normal fibroblasts by the staining pattern of the cell population: reduced nuclear XPF-ERCC1 and the presence of cells in which the heterodimer was exclusively cytoplasmic. This was true irrespective of the antibody used for analysis [monoclonal 3F2 (Cancer Research UK), monoclonal Ab-1 (Neomarkers), or polyclonal anti-XPF (Erasmus Medical Centre)] (data not shown). This indicates that mislocalization of XPF in the cytoplasm is a common consequence of *XPF* mutation.

This raised the possibility that misfolding of mutant XPF caused abnormal subcellular localization of the protein. Therefore, next we asked if these mutant XPF proteins still interacted with their obligate binding partner ERCC1. ERCC1 was detected in the nucleus of normal (wt) fibroblasts, as expected. However, like XPF, ERCC1 was frequently detected in the cytoplasm of *XPF* mutant cells (Figure 2B). All eight *XPF* mutant cells lines tested were readily distinguished from wt cells by their ERCC1 staining pattern. Furthermore, there was a strong correlation between the staining pattern for ERCC1 and XPF in all cell lines. This observation indicates that ERCC1 can interact with each of these mutant XPF proteins. Furthermore, the results suggest that normally ERCC1 enters the nucleus as a heterodimer with XPF, and is retained in the cytoplasm with XPF when XPF is misfolded.

To rule-out the possibility that the abnormal subcellular localization of XPF-ERCC1 was an artifact of immunofluorescence, wt and XPF^R153P^ fibroblasts were fractionated and XPF-ERCC1 was detected by immunoblot in the nuclear and cytoplasmic fractions (Figure 2C). In normal cells (C5RO), XPF-ERCC1 is predominantly nuclear. XPF-ERCC1 was also detected in the cytoplasmic fraction, but to an extent that could be attributed to nuclear contamination, as determined by immunodetection of the nuclear protein nucleophosmin. In contrast, substantially more XPF and ERCC1 were detected in the cytoplasm than in the nucleus of XP51RO cells. Similar results were obtained for other *XPF* mutant cell lines (XP24BR, XP26BR and XP32BR) and using other antibodies against XPF and ERCC1 (data not shown). Degradation products of both ERCC1 and XPF are commonly detected on immunoblot, but were not overrepresented in the mutant cells. These data confirm the immunofluorescence data and support the conclusion that mislocalization of XPF-ERCC1 in the cytoplasm occurs in *XPF* mutant cells.

Remarkably, cytoplasmic XPF-ERCC1 is not detected by immunofluorescence in all *XPF* mutant cells within a population. To quantify the phenomena, the fractions of cells with exclusively nuclear, exclusively cytoplasmic, or pancellular XPF-ERCC1 were determined from immunofluorescence images (Figure 2D). In wt fibroblasts, 93% of cells have XPF-ERCC1 only in the nucleus. Seven percent of cells show pancellular XPF-ERCC1. But never is the complex seen exclusively in the cytoplasm. In all of the *XPF* mutant cell lines, XPF-ERCC1 was detected exclusively in the cytoplasm of a fraction of cells ranging from 3–46% of the total population. Thus all known *XPF* mutations lead to a reduction in nuclear XPF-ERCC1 and an increase in the amount of the complex detected in the cytoplasm.

### Direct detection of XPF^R153P^

**Figure 3:**
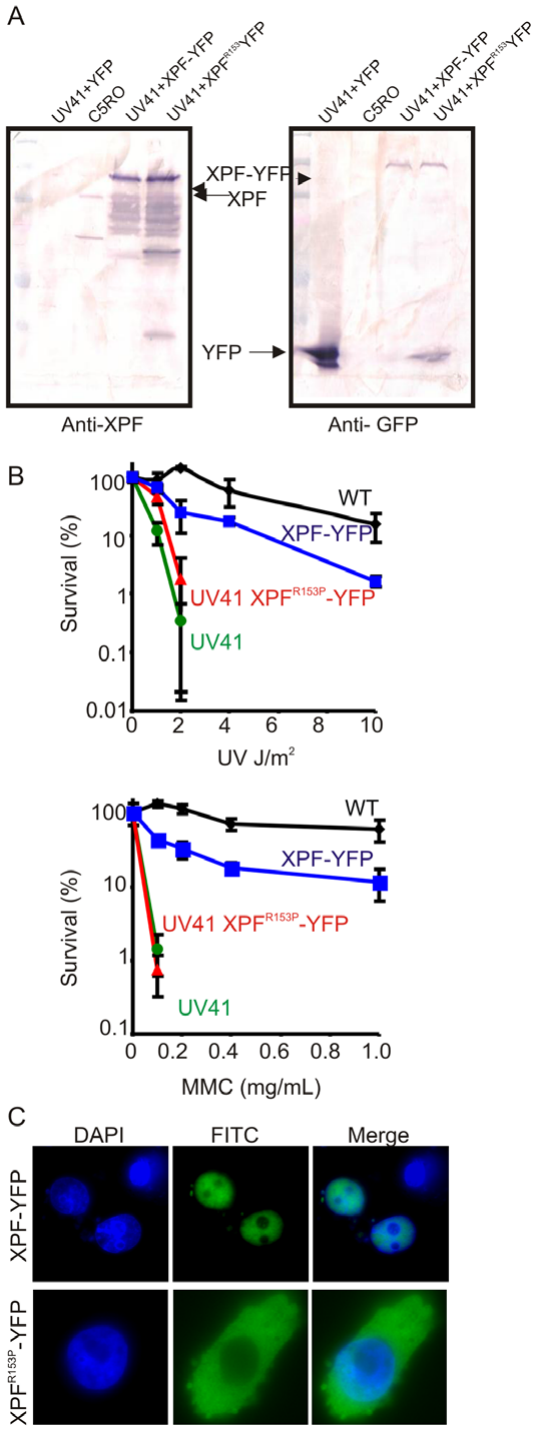
Characterization of XPF-YFP and XPF^153^-YFP in CHO cells. (A) Western blot analysis of XPF-YFP expressed in *Xpf* mutant cells. XPF-deficient hamster cell line, UV41, was transiently transfected with wild type *XPF-YFP* or *XPF^153^-YFP* and the fusion proteins were detected using an antibody against XPF or GFP. C5RO was used as positive control for the XPF blot and as a negative control for the GFP blot. UV41 cells transfected with YFP alone was used as a negative control for XPF blot and as a positive control for GFP blot. (B) Clonogenic survival of wild-type (wt), XPF-deficient CHO cell line UV41, and UV41 transfected with wild type XPF-YFP and XPF^153^-YFP after UV and MMC treatment. Colonies were counted 7–10 days after treatment and results are plotted as mean 3 independent experiments. (C) Subcellular localization of wild type XPF-YFP and XPF^153^-YFP after transient transfection in XPF-deficient the CHO cell line UV41 detected by fluorescence microscopy.

To further rule out the possibility that the cytoplasmic XPF-ERCC1 detected was an artifact generated by non-specific antibodies, human XPF^R153P^ and XPF^WT^ were tagged with YFP and expressed in *Xpf* mutant hamster cells (UV41) for direct detection of XPF protein. The expression of fusion proteins was confirmed by immunoblot using antibodies against human XPF and GFP (Figure 3A). Immunodetection of XPF revealed overexpression of both fusion proteins relative to endogenous XPF protein levels in normal fibroblasts (C5RO). Numerous breakdown products of XPF were also observed, likely due to its overexpression. But only a single fusion protein migrating at the expected molecular mass of full length XPF-YFP was detected using an antibody that detects GFP. To determine if the fusion proteins were functional, transiently transfected cells were tested for their sensitivity to UV to measure NER and mitomycin C (MMC) to measure interstrand crosslink repair (Figure 3B). Wild-type XPF-YFP yielded near complete correction of the hypersensitivity of UV41 mutant cells to UV and MMC. By contrast, despite the fact that XPF^R153P_^YFP was overexpressed to the same extent as XPF^WT^-GFP, this protein was unable to correct either DNA repair defect (Figure 3B), as expected based on the hypersensitivity of the XP51RO patient cell lines [32]. To determine the sub-cellular localization of XPF^R153P^, cells expressing the YFP-tagged protein were plated on glass coverslips and the protein detected by fluorescence microscopy (Figure 3C). XPF^WT^-YFP was exclusively in the nucleus. However, XPF^R153P_^YFP was detected in the cytoplasm of 95% of the transfected cells. This confirms the immunodetection data indicating that mutant XPF is cytoplasmic.

### Characterization of XPF^R153P^-ERCC1 activity in living cells

Unscheduled DNA synthesis (UDS) measures the incorporation of radiolabeled nucleotides into the genome of non-S phase cells after exposure to UV radiation and is a direct measure of NER [35]. Previously, UDS in cells from patient XP42RO (XPF^R799W^) and XP51RO (XPF^R153P^) was reported to be 20% and <5% of that in normal fibroblasts, respectively (Table 1). UV-induced UDS was measurable in all of the mutant *XPF* cell lines except XP51RO. This demonstrates that all of the mutant XPF proteins, with the exception of XPF^R153P^, retain catalytic activity in vivo.

**Table 1:** Characteristics of *XPF* mutant cell lines.

Patient	Mutation Allele 1	Mutation Allele 2	Age (yr)	Skin Cancer	Clinical features	UDS	UV sensitivity	% of cells with non-nuclear XPF-ERCC1	Ref
**C5RO**	none	none		–	normal	100%	1X	7%	[33]
Father of **XP42RO**	R799W	none		–	photosensitivity without skin lesions	100%	1X	rare	[33]
**XP23OS**	455fs	?*	45	–	mild XP	45%	4X	rare	[60]
**XP7NE**	P379S	silent	28	–	mild XP	30%	2X	27%	[61]
**XP62RO°**	R799W	R799W			mild XP with late onset neurodegeneration	20%	not reported	65%	
**XP42RO°**	R799W	R799W	62	+	mild XP with late onset neurodegeneration	20%	2X	33%	[33]
**XP2YO**	T567A ?♦	657fs	65	+	mild XP	17%	3X	n.d.	[46]
**AS871**	R589W	del exon3			severe XP with neurodegeneration	15%	2X	59%	
**XP26BR**	R799W	R799W			mild XP	15%	not reported	33%	
**XP32BR**	R589W	P379S	12	–	mild XP	10%	2X	39%	
**XP24BR**	R799W	R589W	29	–	severe XP with neurodegeneration	5%	3X	74%	[61]
**XP24KY**	R799W	537fs + 7bp	50	–	XP with late onset neurodegeneration	7%	3X	45%	[46]
**XP51RO**	R153P	R153P	16	–	neurodegeneration severe progeria	<5%	10X	>33%	[30]

UDS unscheduled DNA synthesis

*The patient had normal levels of *XPF* transcript, suggesting one allele encodes a full-length mRNA.

**♦:** Mutation could not be confirmed on genomic DNA.

° Siblings.

n.d.  =  not determined

**Figure 4:**
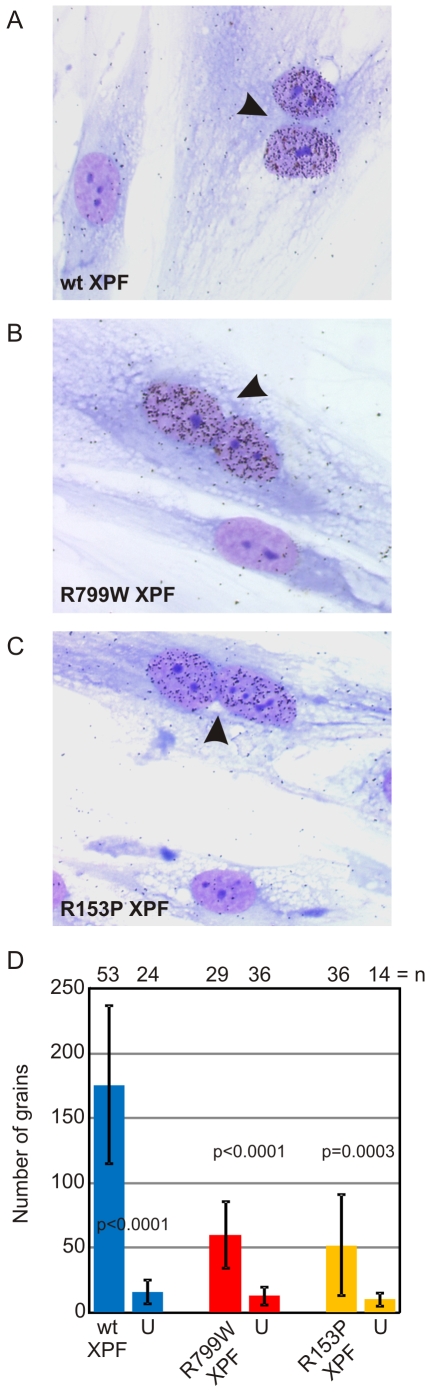
Correction of *XPF* mutant cell NER defect by microinjection of XPF-ERCC1. Primary fibroblasts from XFE progeroid patient XP51RO were fused to create homopolykaryons by treatment with inactivated Sendai virus then plated on glass coverslips. Only homopolykaryons were injected with recombinant XPF-ERCC1 protein complex (A) wild-type (B) XPF^R799W^-ERCC1 (C) XPF^R153P^-ERCC1. The cultures were irradiated with 10 J/m^2^ UV-C and ^3^H-thymidine was added to the culture. UV-induced unscheduled DNA synthesis was detected by autoradiography. Homopolykaryons are indicated with arrows. (D) Histogram indicating the average number of radiographic grains in nuclei injected with each of the recombinant protein complexes and uninjected cells in the same sample. Error bars indicate the standard deviation. N indicates the number of nuclei analyzed in each population. P values for the comparison between injected and uninjected cells were calculated using an unpaired two-tailed Student's t-test.

To ask if XPF^R153P^ is also catalytically active in vivo, recombinant, purified XPF^R153P^-ERCC1 was microinjected into the nuclei of NER-deficient XP51RO primary fibroblasts to determine if UV-induced UDS could be restored. XP51RO cells were first fused on slides by treatment with inactivated Sendai virus to produce homopolykaryons (multinucleate cells). Only homopolykaryons were injected with protein, to permit identification of those cells that were injected with protein. The slides were irradiated with 10 J/m^2^ UV-C, cultured in the presence of ^3^H-thymidine and nuclear grains indicating sites of thymidine incorporation in non-S phase cells measured (Figure 4). As expected, delivery of XPF^WT^-ERCC1 to the nuclei of cells led to a significant increase in the number of grains detected in homopolykaryons relative to individual cells in the same culture (Figure 4A). A significant increase in UV-induced UDS was also detected in homopolykaryons injected with XPF^R153P^-ERCC1 (Figure 4C). This confirms the *in vitro* activity data and establishes that XPF^R153P^ is catalytically active *in vivo* if it is delivered to the nucleus. UDS levels were not recovered to the same extent as when WT protein was injected (Figure 4D). But injection of XPF^R153P^-ERCC1 or XPF^R799W^-ERCC1 led to a similar increase in UDS. This suggests that the incomplete recovery of DNA repair is due to decreased concentration of the recombinant mutant proteins relative to XPF^WT^-ERCC1 (Figure 1). These data support the conclusion that mislocalization of XPF-ERCC1 contributes to the DNA repair defect and symptoms caused by *XPF* mutations.

### Clinical correlation

To determine if the severity of disease associated with a particular mutation in *XPF* could be predicted by the amount of XPF-ERCC1 detected in cell nuclei, the results in Figure 2D were compared to the clinical information available about the patients from which the cells were derived. In Table 1, the cell lines are listed in order of decreasing UDS. Patients with mild disease tend to have greater UDS or DNA repair. Patients XP32BR and XP26BR could be exceptions, but they are too young to know the full extent of their disease. In Figure 2D, the cell lines are clustered into those from patients in which severe disease/neurodegeneration was documented (right) or not yet observed (left). There is a trend towards those with severe disease to have more cells with non-nuclear XPF-ERCC1, but this trend did not reach significance (*p* = 0.06, unpaired Student's t-test), likely due to the small sample size. Therefore, the detection of cells with cytoplasmic XPF-ERCC1, while maybe useful to screen for patients with *XPF* mutations is not sufficient to predict patient prognosis.

## Discussion

Classically, inherited mutations in a gene are associated with a single disease. However, mutations in several genes involved in the NER pathway can result in more than one disease. The most prominent example is *XPD*, which if mutated can cause the cancer-prone disease XP but also Cockayne Syndrome (CS) characterized by photosensitivity, growth retardation, developmental abnormalities and profound neurodegeneration, as well as trichothiodystrophy (TTD), which is similar to CS, but also involves the skin and nails [36]. Similarly, mutations in *XPB* can also cause XP, TTD and a combined XP-CS [37] and mutations in *XPG* can lead to XP or XP-CS [38]. Of all the genes whose products are required for NER, only *XPB*, *XPD* and *XPG* are required for the proper function and stability of the basal transcription factor TFIIH [39]. Thus the more severe symptoms of CS and TTD are attributed to a combined defect in NER as well as transcription [36], [40]–[42]. Mutations in *XPF* were recently linked to a second disease in addition to XP, a disease of systemic accelerated aging termed XFE progeroid syndrome [32]. In this study, we sought to determine how mutations in *XPF* can lead to such a wide variety of symptoms.

### Mutations in *XPF* do not ablate catalytic activity

Since CS and TTD are attributed to defects in transcription [34],[43], the prediction is that mutations in *XPB*, *XPD* or *XPG* that cause CS or TTD should affect basal transcription in addition to NER, whereas mutations that affect only NER cause XP. Indeed, mutations in the catalytic domain of *XPG*, for example A^792^V, disrupt the endonuclease activity of XPG, but not its interaction with TFIIH and therefore causes classical XP [44]. Similarly, a mutation in the helicase domain of XPD, D^234^N, affects NER, but not basal transcription and therefore leads to XP [45]. By analogy, we examined the enzymatic activity of XPF^R153P^ and XPF^R799W^, which cause XFE progeroid syndrome and XP respectively, and discovered that neither mutation ablates the catalytic activity of the protein. Recombinant protein complexes harboring either mutation are able to incise a stem-loop substrate in vitro (Figure 1) and to restore NER *in vivo* (Figure 4). This is in keeping with the fact that patients with XP-F have residual UDS or NER (Table 1). Intriguingly, XP-F patients tend to have much milder photosensitivity and later onset skin cancer than XP patients from other complementation groups with the same level of UDS [46]. One explanation for this is that NER occurs in XP-F cells but at a much slower rate [47], making UDS a relatively poor reflection of the true DNA repair capacity of a cell. In total, these data provide clear evidence that viable mutations in *XPF* do not ablate catalytic activity of the XPF-ERCC1 nuclease. Of note, all XP patients for which the mutation in *XPF* was confirmed by sequencing genomic DNA harbor one of three recurrent point mutations (R799W, R589W or P379S). The rarity and limited repertoire of only hypomorphic point mutations in patients strongly suggests that XPF-ERCC1 nuclease activity is essential for normal embryonic development.

### Stability of XPF^R799W^-ERCC1 and XPF^R153P^-ERCC1 is reduced

Mutations in a single gene could lead to diverse clinical outcomes if mutations differentially affect the stability of the gene product. For example, mutations affecting the stability of the TFIIH complex are linked with TTD but not XP [34]. Total cellular XPF and ERCC1 are dramatically reduced in cells from a patient with XFE progeroid syndrome (Figure 2C). However, XPF levels are reduced to the same extent in whole cell extracts from a patient with mild disease and 20% of the normal level of NER (Figure 1C). Therefore, mutations in *XPF* clearly affect protein level, which undoubtedly contributes to reduced DNA repair and disease. However, the level of XPF-ERCC1 in patient cells, as detected by immunoblotting, is inadequate to explain the differences in the severity in the DNA repair defect and disease between patients with different mutations in *XPF*. Interestingly, in at least a subset of XP-F patients, *XPF* mRNA levels are normal, but XPF protein level is low [46], indicating that mutant XPF is unstable.

### Mutations in *XPF* affect protein subcellular localization

The novel and unexpected finding is that mutation in *XPF* leads to increased cytoplasmic localization of the XPF-ERCC1 nuclease complex (Figure 2 and Figure 3) and that this aberrant subcellular localization is what prevents XPF-ERCC1 from participating in DNA repair (Figure 4). This was demonstrated by immunofluorescence detection of the complex using multiple antibodies. The results were confirmed by examining the subcellular localization of fluorescently tagged recombinant XPF (Figure 2). In further support of this, ERCC1 is also mislocalized to the cytoplasm of cells from the one patient reported with a mutation in *ERCC1* [48].

Cytoplasmic localization of XPF-ERCC1 is not observed in all cells harboring *XPF* or *ERCC1* mutations, suggesting the possibility that mutations affect proper folding of XPF-ERCC1 and that misfolded proteins are preferentially sequestered in the cytoplasm through interactions with other proteins or preferentially degraded. Alternatively, there may be tremendous selection for cells with nuclear XPF-ERCC1. This is consistent with the notion that the repair complex is essential for viability. Indeed, continuous passaging of XP51RO cells over years leads to a striking increase in the fraction of cells with nuclear XPF-ERCC1 and reduced sensitivity to the crosslinking agent mitomycin C (Ahmad, Bhagwat and Niedernhofer, unpublished data). Thus the fraction of cells with cytoplasmic XPF-ERCC1 may be underrepresented in Figure 2D, although only early passage cells were used in this study.

### Disease caused by protein mislocalization

Many human diseases are caused by misrouting or mislocalization of proteins, ranging from metabolic disorders to cancer. Mislocalization of the tumor suppressors p53 [49], FOXO [50], p27^Kip1^ [51] and β-catenin [52] into the cytoplasm rather than the nucleus, leads to a loss of protein function and is associated with cancer. In contrast, mislocalization of NF-κB [53], BRCA1 and BARD1 [53],[54] from the cytoplasm into the nucleus is also associated with a variety of tumors. A classic example of a disease caused by protein mislocalization is cystic fibrosis which is caused by retention of the cystic fibrosis transmembrane conductance regulator (CFTR) protein in the endoplasmic reticulum, instead of its localizing to the cell surface [55],[56]. In addition, nephrogenic diabetes inspidus, retinitis pigmentosa, emphysema and α1-antitrypsin deficiency liver disease are also caused by mislocalized proteins [55].

Mislocalization of proteins may result from a mutated nuclear localization sequence (NLS) or nuclear export sequence (NES). Remarkably, the majority of the missense mutations in *XPF* is at arginine residues and leads to conversion of the arginine to a noncharged residue. So these mutations could affect a complex NLS. All of the point mutations (R→P, R→W and P→S) are also predicted to alter protein structure, supporting the notion that *XPF* mutations affect protein folding and/or protein:protein interactions that are critical for nuclear localization.

Our data add XPF to the list of proteins that if mislocalized contribute to disease. While this leads to novel insight into the regulation of XPF-ERCC1 and DNA repair in cells, the extent of XPF-ERCC1 mislocalization, as measured by immunodetection, does not predict the level of NER (UDS) or disease severity (Table 1). This could be because each mutation differentially affects folding of the protein and thereby differentially affects protein expression, protein degradation and/or cellular localization. Another possibility is that there are modifier proteins that influence disease severity, in particular in patients with homozygous mutations. However, we believe the former is of primary importance based on the observation that titration the level of expression of ERCC1-XPF in mice directly impacts lifespan and the severity of symptoms [57],[58].

In the case of *XPF*, it is the absence of XPF and its binding partner ERCC1 in the nucleus leading to reduced repair of genomic DNA that is disease-causing, rather than toxicity of mislocalized protein. Our data illustrate a novel mechanism by which the DNA repair capacity of a cell is determined: by nuclear localization of XPF-ERCC1. The identification of proteins that regulate this could lead to novel targets for improving DNA repair to treat patients with mutations in *XPF* or reduce cancer risk after exposure to genotoxic agents. Alternatively, these proteins would be excellent targets for small molecule inhibitors that would reduce repair and thereby prevent tumor resistance to genotoxic cancer therapies.

## Materials and Methods

### Biochemical characterization of XPF^R153P^-ERCC1 and XPF^R799W^-ERCC1

Purification of recombinant XPF–ERCC1 was performed essentially as previously reported [27] from baculovirus-infected Sf9 insect cells using a His_6_ tag on ERCC1. In brief, plasmids pFastBac1-*XPF* and pFastBac1-*ERCC1*-His were used to transfect Sf9 insect cells, and to amplify the virus according to the manufacturer's instructions (BAC TO BAC system; Life Technologies). Cell extracts were prepared 65 hr after infection with an MOI of 5 and highly purified protein was obtained using chromatography on Ni−agarose, gel-filtration and heparin columns. Only XPF-ERCC1 eluting as proper heterodimer on the gel filtration column at ∼65 ml of eluant was collected. The aggregated protein, eluting in the void volume (∼40–50 ml), was not used in experiments.

The endonuclease activity of wild-type and mutant XPF-ERCC1 was performed using a stem−loop substrate also as previously described [27]. A stem12−loop22 oligonucleotide (GCCAGCGCTCGGT_22_CCGAGCGCTGGC) was 5′-^32^P end-labeled. Nuclease reactions were performed on 100 fmol of DNA substrate and 20–200 fmol of XPF-ERCC1 protein in a total volume of 15 µl in optimized nuclease buffer (25 mM HEPES pH 8.0, 40 mM NaCl, 10% glycerol, 0.5 mM β-mercaptoethanol, 0.1 mg/ml bovine serum albumin and 0.4 mM MnCl_2_ or 2 mM MgCl_2_). The reactions were incubated at 30°C for 2 h and stopped by adding 15 µl of 90% formamide/10 mM EDTA and heating at 95°C for 5 min. Samples were loaded onto 15% denaturing polyacrylamide gels and reaction products were visualized by autoradiography and quantified on a PhosphorImager (STORM860; Molecular Dynamics).

### Cell lines and culturing

Human fibroblasts immortalized with hTert were cultured in Ham's F10 with 10% fetal calf serum and antibiotics and incubated at 3% oxygen as described previously [32]. Cell lines included those derived from a normal individual (C5RO) [59], the parent of a patient, heterozygous for a mutation in *XPF* [33], XP-F patients (XP42RO) [33], XP23OS [60], XP24KY [46], XP7NE [61], XP32BR, XP26BR, XP24BR [61], and XP62RO, and a patient with XFE progeroid syndrome caused by a mutation in *XPF* (XP51RO) [32]. Unscheduled DNA synthesis (UDS) in these cells lines was previously reported as referenced above and confirmed in mixed cultures (XP-F cells co-cultured with normal cells using a more accurate click-staining method, as recently described [62].

### Immunodetection of XPF in patient cells

Cells were trypsinized, washed twice with PBS and lysed with 1 ml NETT buffer (100 mM NaCl, 50 mM Tris base pH 7.5, 5 mM EDTA pH 8.0, 0.5% Triton X-100) containing Complete™ mini protease inhibitor cocktail and phosphatase inhibitor cocktail (Roche Molecular Biochemicals). Then the lysates were freeze-thawed twice in liquid nitrogen to disrupt nuclear membranes. From each sample, 50 µg of protein was resolved on 10% SDS-PAGE gels after boiling for 10 min in the presence of loading buffer. XPF was detected using a human XPF monoclonal antibody (clone 219; Neomarkers, Fremont, CA) at a dilution of 1∶1000.

### Differential immunofluorescence of fibroblasts isolated from patients with mutations in *XPF*

Cultures of primary human fibroblasts from patients with mutations in *XPF* or a normal individual were grown in the presence of different size beads (2 µm or 0.8 µm; Sigma). After 24 hr the cultures were trypsinized and washed extensively with phosphate-buffered saline to remove any extracellular beads. The cells were then mixed in various combinations and co-plated on glass coverslips to provide internal controls of normal XPF-ERCC1 protein levels [34]. After 16 hr, the cells were fixed with 2% paraformaldehyde in sodium phosphate buffer, pH 7.4, for 15 min then permeabilized with 0.1% Triton X-100 in PBS. The samples were immunostained with polyclonal anti-ERCC1 (1∶2000; [63]) or polyclonal anti-XPF (1∶1000; [16]) followed by goat anti-rabbit ALEXA 488 (1∶500; Molecular Probes). Samples were stained with Dapi to identify nuclei and examined by phase contrast microscopy to identify the genotype of the cells according to their bead content and by fluorescence microscopy for immunodetection of repair proteins.

#### Cell fractionation

Cells were fractionated into nuclear and cytosolic fractions as described [64], with minor modification. In brief, cells were trypsinized, pelleted and washed twice with PBS. The pellet was vortexed at maximum speed for 15 sec with 200 µl of CERI reagent from the Pierce NE-PER fractionation kit (Pierce Biotech) with Complete™ mini protease inhibitor cocktail, then incubated on ice for 10 min. This was followed by addition of 11 µl of CERII, vortexing for 5 sec and incubation on ice for 1 min. The mixture was spun at 13,000 rpm for 5 min and the supernatant collected as the cytosolic fraction. The nuclei in the pellet were suspended in extraction buffer (15 mM Tris-HCl pH 7.3, 1 mM EDTA, 0.4 M NaCl, 1 mM MgCl_2_, 10% glycerol, 10 mM β-mercaptoethanol and Complete™ mini protease inhibitor cocktail), mixed for 1 hr at 4°C and spun down at 13,000 rpm for 10 min. The supernatant was collected as the soluble nuclear fraction.

### Subcellular localization of XPF–YFP in Chinese hamster Ovary (CHO) cells

*XPF* cDNA was cloned into pYFP-N1 (BD Biosciences Clontech, Palo Alto, CA) such that YFP was expressed as fusion protein at the C-terminus of XPF. This construct, pXPF-YFP-N1, was then used to create XPF^R153P^-YFP by QuickChange^R^ Site-Directed Mutagenesis Kit (Stratagene, Cedar Creek, TX) according to the manufacturer's instructions. The wild type and mutant constructs were transfected in XPF-deficient CHO cell lines UV41 or UV47 using lipofectamine 2000 (Invitrogen, Carlsbad, CA) following the manufacturer's instructions. Cells expressing YFP were flow sorted using Dako Cytomation MoFLo high-speed cell sorter (Dako North America, Carpinteria, CA) 24–48 hrs after transfection.

To study the subcellular localization of XPF, YFP-positive CHO cells were plated on glass coverslips and grown to 95% confluency. The next day, the samples were fixed with 2% paraformaldehyde in sodium phosphate buffer, pH 7.4, for 15 min. The cells were permeabilized with 0.1% Triton X-100 in phosphate-buffered and nuclei were stained with Dapi-vector shield (Vector Laboratories, Inc. Burlingame, CA). XPF-YFP was visualized using an Olympus BX51 fluorescent 4 microscope at 60–100X magnification.

### Clonogenic survival assays of wild-type and mutant CHO cells

Wild type (AA8), XPF-deficient (UV41), XPF-YFP and XPF^R153P^-YFP cells were seeded in 6 cm dishes in triplicate at 10^3^–10^4^ cells per plate, depending on the dose of genotoxin. After 16 hr, the cells were irradiated with UV-C or exposed to mitomycin C (MMC). After approximately one week, the cultures were fixed and stained with 50% methanol, 7% acetic acid and 0.1% Coomassie blue. Colonies, consisting of at least 10 cells, were counted using a Nikon SMZ 2B 15 stereomicroscope microscope with 10X eyepiece. The data were plotted as the number of colonies that grew on the treated plates relative to untreated plates ± the standard error of the mean for 2–3 independent experiments.

### Immunoblotting of XPF in Wt and mutant CHO cells

Whole cell extracts were prepared from C5RO and UV41 cells transfected with vectors expressing YFP, XPF-YFP or XPF^R153P^-YFP. Proteins were separated by SDS PAGE using a 10% gel. XPF was detected using a human XPF monoclonal antibody (clone 219; Neomarkers, Fremont, CA) at a dilution of 1∶1000. YFP was detected using a GFP monoclonal antibody (Clones 7.1 and 13.1; Roche, Indianapolis, IN) at a dilution of 1∶1000.

### Correction of *XPF* mutant cell UV sensitivity by micro-injection of recombinant XPF-ERCC1

Microinjection of purified proteins was performed as previously described [65],[66]. Briefly, primary human fibroblasts from XP51RO were fused by treating cultures with inactivated Sendai virus and then plated on glass coverslips. Subsequently, purified, recombinant XPF-ERCC1 protein complex (wild type or containing the R799W or R153P substitution in XPF) was injected into the nuclei of homopolykaryons. The cultures were irradiated with 10 J/m^2^ UV-C and pulse labeled for 3 hrs with ^3^H-thymidine. Unscheduled DNA synthesis (UDS) was detected by autoradiography.

One to ten femtoliters of a 10–100 nM solution was injected into the nuclei of 10–20 homopolykaryons for each of the three recombinant proteins and the number of radiographic grains counted in at least 20 nuclei of the homopolykaryons and a similar number of nuclei of single cells in the same sample. The mean and standard deviation of the number of grains was calculated for each of the three proteins. An unpaired, two-tailed Student's t-test was used to determine if there was a significant difference in unscheduled DNA synthesis between cells that were injected with recombinant XPF-ERCC1 and cells that were not injected.
